# Clinical symptoms associated with prehospital delay in first-ever acute stroke

**DOI:** 10.17305/bb.2026.14308

**Published:** 2026-06-05

**Authors:** Hatice Yelda Yıldız, Emine Işıl, Tuğba Yurdakul, Özgür Canpolat, Doğan Kaya, Ferda Kaya Zaman, Yakup Krespi

**Affiliations:** 1 Department of Neurology, Faculty of Medicine, Istinye University, İstanbul, Türkiye; 2 Department of Internal Medicine, Prof. Dr. Cemil Taşçıoğlu City Hospital, İstanbul, Türkiye; 3 Department of Neurology, Prof. Dr. Mazhar Osman Mental and Nervous Diseases Hospital, İstanbul, Türkiye; 4 Department of Family Medicine, Republic of Turkey Ministry of Health, İstanbul Haydarpaşa Numune Training and Research Hospital, İstanbul, Türkiye; 5 Department of Family Medicine, Merter Keresteciler Family Health Center, İstanbul, Türkiye; 6 Department of Family Medicine, Memorial Bahçelievler Hospital, İstanbul, Türkiye

**Keywords:** Acute stroke, prehospital delay, hospital arrival time, stroke symptoms, stroke awareness, intravenous thrombolysis, endovascular treatment, stroke outcome

## Abstract

Early hospital arrival is essential for timely reperfusion therapy in acute stroke. This study aimed to identify clinical, symptom-related, and prehospital factors associated with delayed hospital arrival among patients with first-ever acute stroke, with particular focus on the 4.5-hour therapeutic window. This prospective single-center observational study was conducted at a comprehensive stroke center in Istanbul, Türkiye, between December 2023 and October 2024. Among 362 patients screened for suspected acute cerebrovascular events, 205 adults with first-ever acute ischemic stroke, intracerebral hemorrhage, or transient ischemic attack managed through the acute stroke pathway were included. Data were collected using a structured clinical form and a questionnaire on barriers to accessing acute stroke treatment, administered through face-to-face interviews with patients’ relatives. Prehospital delay was defined as the interval from last known well (LKW) to hospital arrival. Patients were analyzed according to arrival within 1 hour, 2 hours, 3 hours, and 4.5 hours, using appropriate comparative statistical tests. The mean LKW-to-arrival time was 338.66 ± 345.67 minutes, with a median (IQR) of 240 (90–720) min. Overall, 29.3% of patients arrived within 1 hour, 41.0% within 2 hours, 48.3% within 3 hours, and 58.5% within 4.5 hours. Facial droop was consistently associated with earlier hospital arrival across multiple time thresholds (*P* ≤ 0.004), and syncope was more frequent among early presenters (*P ═* 0.001). Conversely, visual symptoms were associated with delayed presentation, including vision loss after 3 hours and diplopia beyond 4.5 hours (*P ═* 0.042 for both). Diabetes mellitus was associated with delayed arrival at the 1-hour and 2-hour thresholds, while hypertension was more common among patients arriving after 4.5 hours. Prehospital delay remains a substantial barrier to timely acute stroke care. Recognizable symptoms such as facial droop may facilitate earlier presentation, whereas less typical symptoms, particularly visual disturbances, may contribute to delayed arrival. These findings support locally tailored public awareness strategies and optimized prehospital stroke pathways that emphasize both typical and atypical stroke symptoms.

## Introduction

Acute stroke remains a leading cause of death and long-term disability globally, with the efficacy of acute reperfusion therapies being profoundly time-dependent [1]. Population-based epidemiological studies underscore the substantial burden of cerebrovascular disease, reporting cumulative incidence rates of up to 218 per 100,000 in men and 127 per 100,000 in women within European cohorts, thereby emphasizing the significant healthcare impact of acute stroke [2]. In ischemic stroke, irreversible neuronal injury commences within minutes of arterial occlusion. The concept of “time is brain” has been quantitatively demonstrated, with an estimated loss of 1.9 million neurons per untreated minute. This biological urgency necessitates immediate recognition, rapid transportation, and early initiation of reperfusion therapy in eligible patients.

The effectiveness of acute stroke treatment hinges not only on the availability of intravenous thrombolysis (IVT) and endovascular therapies (EVTs) but also on patients’ ability to access appropriate care within a narrow therapeutic window. While in-hospital stroke pathways have improved over time, prehospital delay consistently accounts for the largest proportion of total treatment delay, leading many patients to arrive too late to benefit from evidence-based acute interventions [3–5]. Consequently, optimizing the prehospital stroke chain has become a central priority in modern stroke care systems, with an emphasis on rapid symptom recognition, prompt emergency medical service (EMS) activation, prehospital notification, and timely transport to stroke-ready centers [6, 7]. Furthermore, recent evidence highlights the importance of region-specific organizational strategies to mitigate geographic disparities in access to acute stroke treatment. These strategies include optimized EMS routing algorithms, coordinated interfacility transfer systems, and regional stroke network planning tailored to local healthcare infrastructure and travel distances [8].

Despite advancements in public education and emergency stroke organization, prehospital delay persists as a significant challenge across diverse healthcare systems. Prior studies indicate that hesitation in seeking help, inadequate symptom recognition, reliance on non-emergency medical pathways, and variability in symptom presentation all contribute to delayed hospital arrival [9–11]. Public stroke warning campaigns have been developed to address these barriers; however, while some interventions have improved ambulance utilization and reduced delay in selected settings, the overall evidence remains heterogeneous, and the translation of knowledge into rapid patient behavior is suboptimal [12]. Accordingly, current evidence suggests that effective stroke care requires not only technological and organizational readiness but also a more comprehensive understanding of patient-, bystander-, and system-level determinants of delayed presentation [5, 7].

Crucially, the etiologies and patterns of prehospital delay can vary based on regional healthcare infrastructure, sociocultural context, symptom perception, and emergency referral pathways. Therefore, localized data are essential to identify modifiable barriers and guide realistic improvements in stroke organization. This study aimed to investigate factors associated with delayed hospital arrival among patients presenting with first-ever acute stroke, with a particular focus on the critical 4.5-hour therapeutic window from last known well (LKW) to hospital arrival. We further sought to evaluate the relationship of demographic, clinical, symptom-related, and healthcare access variables with prehospital delay to identify locally relevant targets for enhancing timely access to acute stroke care.

## Materials and methods

### Ethical statement

The research adhered to the principles of the Declaration of Helsinki. Ethical clearance was secured from the Human Research Ethics Committee of Istinye University prior to data analysis. The study received approval at the committee meeting held on November 06, 2023 (Protocol No: 2023-350). All participants were thoroughly informed about the study’s purpose, procedures, and voluntary nature before enrollment. Written informed consent was obtained from patients or their legally authorized relatives before participation. The study was conducted prospectively, utilizing structured interviews with patients’ relatives to administer questionnaire-based assessments in accordance with the study protocol. Participant confidentiality and data privacy were maintained throughout the study, conforming to institutional and ethical guidelines.

### Study design and objective

This study was designed to investigate factors associated with delayed hospital arrival among patients experiencing their first-ever stroke, with a specific emphasis on the critical 4.5-hour therapeutic window, measured from the LKW time to hospital arrival. The primary objective was to identify patient-related, socioeconomic, and organizational factors that may contribute to prehospital delay in acute stroke presentation. Early hospital arrival is paramount for eligibility for reperfusion therapies, including IVT and mechanical thrombectomy, which significantly reduce mortality and long-term disability when administered promptly.

### Study setting and timeframe

This prospective observational study was conducted at the Stroke Center of Istinye University Faculty of Medicine Training and Research Hospital in Istanbul, Türkiye. While the center is situated within one of the city’s six healthcare service regions, it also receives referrals from surrounding provinces, serving an estimated population of approximately 5–6 million individuals. The hospital operates as one of eight comprehensive stroke centers in the metropolitan area, comprising six public and two private institutions, managing an estimated 1000–1500 stroke cases annually. Data collection occurred between December 2023 and October 2024.

### Study population

A total of 362 patients presenting with suspected acute cerebrovascular events were initially screened during the study period. Following the application of predefined inclusion and exclusion criteria, including restriction to first-ever stroke presentations, 205 patients were ultimately included in the final study cohort. The study population comprised adult patients (≥18 years) admitted to the stroke center with a diagnosis of acute stroke after emergency department evaluation. Eligible patients included those hospitalized with first-ever acute ischemic stroke, intracerebral hemorrhage, or transient ischemic attack (TIA) following emergency department evaluation and neurological assessment. TIA cases were analyzed within the hyperacute cerebrovascular presentation spectrum due to their initial management through the acute stroke pathway. Patients with a prior history of stroke were excluded to specifically focus on first-ever stroke presentations.

During the study period, all potentially eligible patients presenting to the stroke center were screened consecutively. Patients were excluded if they had a prior history of stroke or non-stroke diagnoses. Functional outcome analyses at 3 months were performed only on patients with available follow-up data. The patient selection and follow-up process are summarized in the study flow diagram (Figure 1).

**Figure 1.**
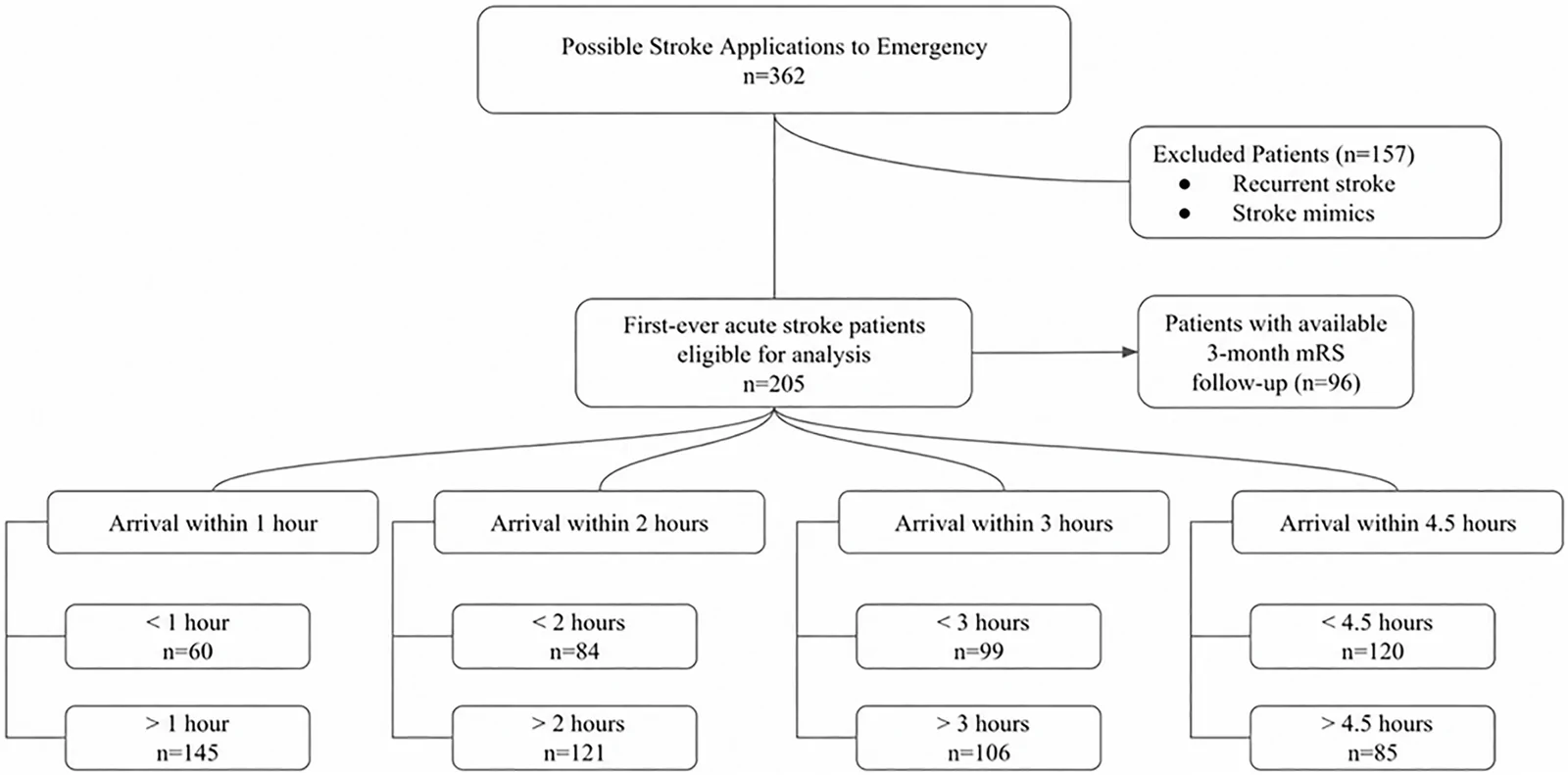
**Flow diagram of patient selection and prehospital delay threshold analysis.** Initial screening identified 362 patients presenting to the emergency department with suspected acute stroke. After excluding recurrent stroke cases and stroke mimics, 205 first-ever acute stroke patients were included in the final study cohort. Patients were then categorized based on predefined clinically relevant arrival time thresholds (defined as time from last known well to hospital arrival: <1 hour, <2 h, <3 h, and <4.5 h). This stratification aimed to investigate factors associated with ultra-early and delayed presentation patterns within the acute stroke pathway.

### Exclusion criteria

Patients were excluded if they had non-stroke diagnoses (e.g., stroke mimics such as epilepsy, metabolic imbalance, migraine, or other structural brain diseases), were admitted through outpatient clinics after the acute phase, had a documented history of prior stroke, lacked an accompanying relative available to complete the survey, or were transferred to another institution or died before data collection was complete.

### Data collection

Data were collected using two instruments. The first was a structured patient information form developed by the research team to record clinical characteristics, demographic characteristics, and time intervals related to symptom onset and hospital arrival.

The second instrument was a structured questionnaire titled “Barriers to Accessing Acute Stroke Treatment,” developed by the research team based on previously published literature evaluating prehospital stroke delay, stroke awareness, and healthcare-seeking behavior. The questionnaire was designed to assess sociodemographic characteristics, symptom recognition, emergency response behavior, transportation pathways, and awareness of acute stroke treatment options.

Structured face-to-face interviews were conducted with patients’ relatives by members of the research team during hospitalization. Relatives were selected as respondents due to their perceived reliability as a source of information regarding prehospital events and decision-making processes. Questionnaire responses were recorded using predefined categorical response options whenever applicable and subsequently grouped into clinically meaningful analytical categories for statistical analysis.

### Questionnaire items

The structured questionnaire, titled “Barriers to Accessing Acute Stroke Treatment,” assessed explanatory variable domains encompassing sociodemographic characteristics, prehospital pathway behaviors, symptom response patterns, EMS utilization, healthcare interactions, and stroke awareness. This questionnaire was administered to patients’ relatives via structured interviews (Table 1).

**Table 1:** Domains and items of the structured questionnaire assessed in the present study

**Domain**	**Items**
Sociodemographic information	1. Who does the patient live with? Who provides care for the patient? 2. Does the patient live alone? 3. What is the patient’s employment status?4. What is the patient’s professional occupation?5. What is the patient’s highest level of education?
Prehospital pathway	1. How did the patient arrive at the hospital? 2. Was medical assistance sought before arriving at the hospital? 3. Was emergency medical service (112) contacted?4. Did the patient first present directly to a stroke center?
Actions taken at symptom onset	1. What actions were taken when the symptoms first appeared?
Healthcare interaction during the prehospital phase	1. Were you given priority evaluation at the first healthcare facility you presented to? 2. Did a healthcare professional inform you that the patient’s condition might be a stroke?
Emergency medical services awareness	1. Did emergency medical service (112) personnel inform you about possible treatment options? 2. Did emergency medical service personnel inform you that thrombolytic therapy could be administered? 3. Did emergency medical service personnel inform you that thrombectomy could be performed? 4. Did emergency medical service personnel explain the importance of time management in stroke?
Stroke awareness	1. Had you previously heard about the importance of thrombolytic therapy or thrombectomy in stroke treatment?

### Primary outcomes

The primary outcome variable was prehospital delay, defined as the time interval between the LKW time and hospital arrival. Variables analyzed included age, sex, educational level, socioeconomic status, stroke awareness, transportation method, stroke severity (National Institutes of Health Stroke Scale [NIHSS]), stroke subtype, clinical stroke syndrome, distance to hospital, decision-making time for seeking medical care, and EMS usage.

Functional outcome at 3 months was evaluated using the modified Rankin Scale (mRS), obtained through outpatient follow-up evaluations or structured telephone interviews conducted by the clinical stroke team. Follow-up assessments adhered to routinely applied institutional stroke follow-up procedures.

For statistical analysis, patients with an mRS score of 0 were categorized as having a favorable outcome, while those with mRS scores of 1–6 were categorized as having an unfavorable outcome. This strict dichotomization strategy was chosen to specifically assess complete functional recovery without residual disability in relation to prehospital delay and symptom presentation characteristics.

### Definitions

LKW was defined as the last time the patient was known to be neurologically normal before the onset of stroke-related symptoms. This information was gathered from the patient, accompanying relatives, EMS records, or referral documentation when available. In cases of unwitnessed symptom onset, such as wake-up strokes, the last known symptom-free time served as the LKW reference point. Cases with highly uncertain or unverifiable onset times were excluded from analyses requiring precise temporal classification.

Hospital arrival time was defined as the first documented arrival at a healthcare facility capable of initiating acute stroke evaluation. For transferred patients, the initial presentation time at the first medical facility was used for prehospital delay calculations, rather than the arrival time at the comprehensive stroke center.

Stroke severity was assessed using the NIHSS. Clinical stroke syndromes were classified according to the Bamford classification (Total Anterior Circulation Infarct [TACI], Partial Anterior Circulation Infarct [PACI], Lacunar Infarct [LACI], Posterior Circulation Infarct [POCI]). Stroke mimics included conditions such as epilepsy, metabolic imbalance, migraine, and other non-stroke structural brain diseases.

### Statistical analysis and tools

Statistical analyses were performed using Wistats v3.0 (WisdomEra Corp., Istanbul, Turkey), which operates on the Python programming language and utilizes statistical libraries including SciPy v1.2.3, scikit-learn v0.24.0, and statsmodels v0.9.0 (https://wistats.wisdomera.io).

Continuous variables were summarized as mean ± standard deviation (SD), while categorical variables were expressed as counts and percentages. Data distribution was assessed using skewness and kurtosis statistics, and normality was evaluated with the Shapiro–Wilk test.

Patients were categorized based on arrival time thresholds of 1 hour, 2 h, 3 h, and 4.5 h. These predefined thresholds were selected to explore potential differences between ultra-early and progressively delayed hospital presentation periods across clinically meaningful stages of acute stroke management and reperfusion eligibility. The analyses were thus designed to characterize exploratory associations across distinct therapeutic windows rather than to develop a single predictive model.

Comparative analyses were conducted to evaluate differences between early and delayed arrival groups at each time threshold. Associations between categorical variables—such as sex, comorbidities (hypertension, diabetes mellitus, atrial fibrillation, coronary artery disease), stroke subtype, and presenting symptoms—were analyzed using the chi-square test or Fisher’s Exact test as appropriate. Fisher’s Exact test was preferentially used for sparse categorical comparisons involving small subgroup counts or zero-frequency cells.

Comparisons involving continuous variables, including age, Glasgow Coma Scale (GCS) score, NIHSS score, and LKW time, were performed using the Independent Samples *t*-test for normally distributed data or the Mann–Whitney *U* test for non-normally distributed variables.

To assess clinical outcomes, patients were also grouped according to 3-month functional status using mRS. Differences between patients with good and poor prognosis were evaluated using the same statistical methods, tailored to variable type and distribution. A two-tailed P-value < 0.05 was considered statistically significant for all analyses.

**Table 2:** Baseline characteristics of the study population

	**cases** **Mean ± SD, *n* (%)**
N	205 (100%)
Age	69.88 ± 13.39
Gender	
female	85 (41.5%)
male	120 (58.5%)
Last known well (minutes)	338.66 ± 345.67; median (IQR): 240 (90–720)
Arrival within 1 hour	
<1 hour	60 (29.3%)
>1 hour	145 (70.7%)
Arrival within 2 hours	
<2 hours	84 (41.0%)
>2 hours	121 (59.0%)
Arrival within 3 hours	
<3 hours	99 (48.3%)
>3 hours	106 (51.7%)
Arrival within 4.5 hours	
<4.5 hours	120 (58.5%)
>4.5 hours	85 (41.5%)
Glasgow Coma Scale	13.92 ± 1.78
NIHSS	8.52 ± 6.39; median (IQR): 7 (3–15)
Oxfordshire community stroke project	
LACI	32 (15.6%)
PACI	85 (41.5%)
POCI	43 (21.0%)
TACI	27 (13.2%)
TIA	18 (8.8%)
Large vessel occlusion	
No	163 (79.5%)
Yes	42 (20.5%)
Transfer to the intensive care unit	
No	185 (90.2%)
Yes	20 (9.8%)
In-hospital death	
No	196 (95.6%)
Yes	9 (4.4%)
Intravenous thrombolysis	
No	156 (76.1%)
Yes	49 (23.9%)
Endovascular treatment	
No	193 (94.1%)
Yes	12 (5.9%)
3-month mRS outcome group	
Good	25 (26.0%)
Poor	71 (74.0%)
Who does the patient live with? Who provides care for them?	
Alone	14 (8.8%)
Other	14 (8.8%)
Spouse	43 (26.9%)
With children and spouse	89 (55.6%)
Living alone	
No	146 (91.2%)
Yes	14 (8.8%)
Currently employed	
No	141 (88.1%)
Yes	19 (11.9%)
Professional status	
Daily worker	1 (0.8%)
Other	69 (53.9%)
Public employee	12 (9.4%)
Self-employed	41 (32.0%)
Worker	5 (3.9%)
Educational status	
High school graduate	16 (13.1%)
Master’s degree	17 (13.9%)
Middle school graduate	11 (9.0%)
Primary school dropout	32 (26.2%)
Primary school graduate	46 (37.7%)
Education group	
Higher	33 (27.0%)
Lower	89 (73.0%)
How did you get to the hospital?	
Direct ambulance (112)	150 (73.2%)
In-hospital transfer	5 (2.4%)
Intercity transfer	34 (16.6%)
Intracity transfer	11 (5.4%)
Walk-in	5 (2.4%)
Medical support sought prior to hospital presentation	
Contacted family physician	1 (0.6%)
Emergency services (112) were called	166 (97.1%)
Presented to neurology outpatient clinic	4 (2.3%)
First arrival at stroke center	
No	45 (22.0%)
Yes	160 (78.0%)
What did you do at the onset of the symptoms?	
Analgesic	1 (0.5%)
Another person’s anticoagulant	62 (30.7%)
Another person’s antihypertensive medication	3 (1.5%)
No treatment administered	107 (53.0%)
Own antihypertensive medication	29 (14.4%)
Were you given priority evaluation at the first hospital you presented to?	
No	11 (7.7%)
Yes	132 (92.3%)
Did the home healthcare team inform you that the patient’s condition was a stroke?	
No	99 (60.4%)
Yes	65 (39.6%)
Did the emergency medical services (112) personnel inform you about possible treatment options?	
No	135 (86.0%)
Yes	22 (14.0%)
Did the emergency medical services (112) personnel inform you that thrombolytic therapy could be administered to the patient?	
No	136 (88.3%)
Yes	18 (11.7%)
Did the emergency medical services (112) personnel inform you that thrombectomy could be performed for the patient?	
No	129 (92.1%)
Yes	11 (7.9%)
Did the emergency medical services (112) personnel explain the importance of time management in ischemic stroke?	
No	120 (78.4%)
Yes	33 (21.6%)
Had you previously heard about the importance of thrombolytic therapy and thrombectomy in stroke?	
No	134 (76.6%)
Yes	41 (23.4%)

Percentages were calculated using available-case denominators for each variable due to missing questionnaire responses and incomplete follow-up data. Continuous variables are presented as mean ± standard deviation and median (interquartile range), where appropriate. **Abbreviations**: IQR, interquartile range; LACI, lacunar infarct; mRS, modified Rankin Scale; N, number; NIHSS, National Institutes of Health Stroke Scale; PACI, partial anterior circulation infarct; POCI, posterior circulation infarct; SD, standard deviation; TACI, total anterior circulation infarct; TIA, transient ischemic attack.

Given that the primary objective of the study was the exploratory characterization of factors potentially associated with prehospital delay across clinically relevant arrival thresholds, the analyses primarily relied on unadjusted comparative statistical methods. The findings should therefore be interpreted as exploratory associations rather than evidence of independent causal relationships. Future studies with larger multicenter cohorts and predefined multivariable models are necessary to confirm these observations.

As the analyses were exploratory and involved multiple subgroup comparisons across different arrival thresholds, the reported *P*-values should be interpreted cautiously, particularly for associations with borderline statistical significance.

Furthermore, since the primary analytical objective focused on clinically predefined therapeutic arrival thresholds rather than continuous temporal prediction, formal continuous time-to-arrival regression modeling was not performed in this exploratory analysis.

## Results

A total of 205 patients diagnosed with acute stroke were included in the analysis. The mean age of the study population was 69.88 ± 13.39 years, with 58.5% being male. The mean GCS score was 13.92 ± 1.78, while the mean NIHSS score at admission was 8.52 ± 6.39, with a median (interquartile range [IQR]) of 7 (3–15). The mean time from LKW to hospital arrival was 338.66 ± 345.67 min. According to arrival times, 29.3% of patients arrived within the first hour, 41.0% within 2 h, 48.3% within 3 h, and 58.5% within the 4.5-hour therapeutic window (Figure 2A). Hypertension was present in 65.5% of patients, diabetes in 37.9%, atrial fibrillation in 17.2%, and coronary artery disease in 28.4%. IVT was administered to 23.9% of patients, and endovascular treatment to 5.9%. At 3 months, 26.0% of patients had no disability (mRS 0), while 74.0% were classified as having a poor prognosis. Detailed baseline characteristics are presented in Table 2. Prehospital pathway characteristics according to early and delayed arrival groups are illustrated in Figure 2B.

**Figure 2.**
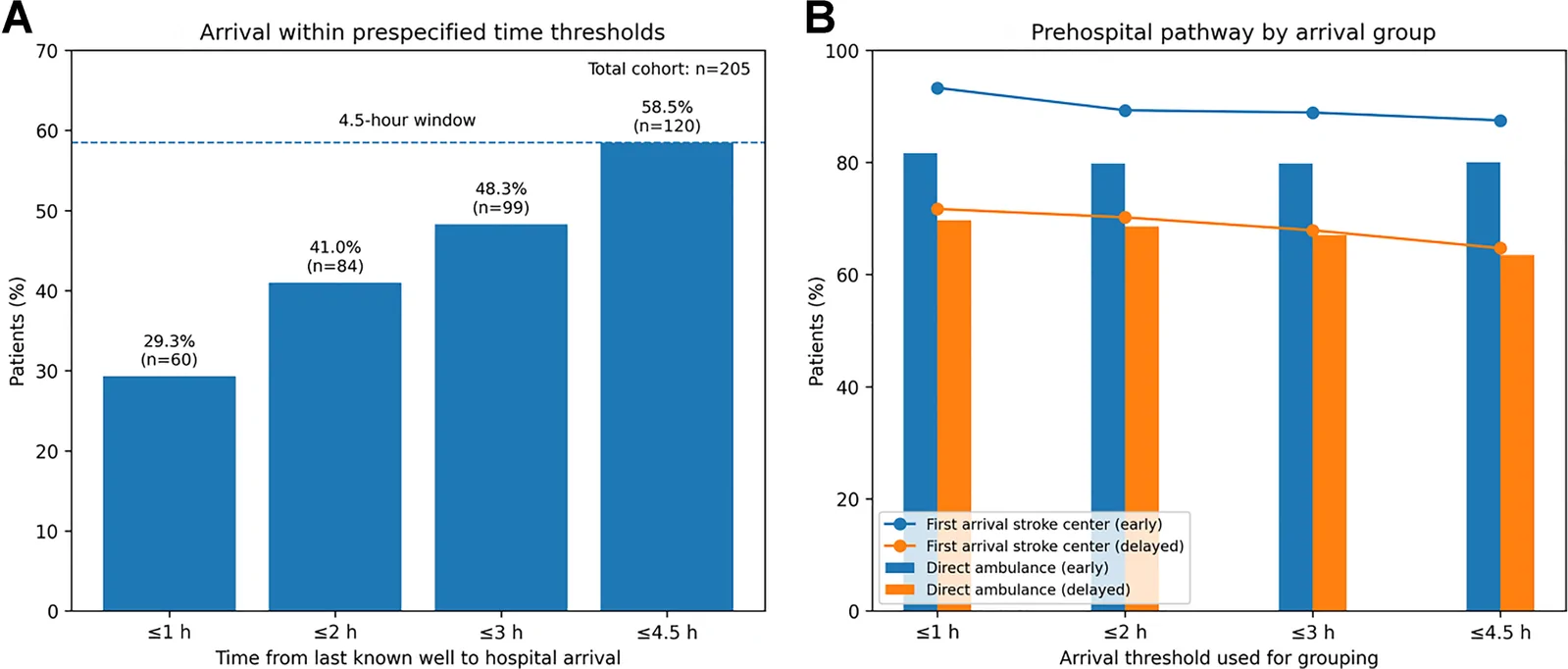
**Arrival timing and prehospital pathway characteristics among patients with first-ever acute stroke.** (A) This panel illustrates the proportion of acute stroke patients arriving at the hospital within specified time thresholds from LKW, specifically ≤1 hour, ≤2 h, ≤3 h, and ≤4.5 h. The dashed horizontal line denotes the upper limit of the standard intravenous thrombolysis eligibility window (4.5 h). (B) This panel presents a comparison of prehospital pathway characteristics between early and delayed arrival cohorts, categorized by the same time thresholds as in Panel (A). Specifically, it details the proportion of patients transported directly by EMS and the proportion of patients whose initial medical contact was established at a designated stroke center. **Abbreviations**: EMS, emergency medical services; LKW, last known well.

### Comparison according to 1-hour arrival time

When patients arriving within 1 hour were compared with those arriving after 1 hour, diabetes mellitus was significantly more frequent among patients with delayed arrival (42.4% vs. 27.1%, *P ═* 0.029). Certain presenting symptoms were also associated with early hospital presentation. Facial droop was significantly more common among patients arriving within the first hour (12.3% vs. 2.2%, *P ═* 0.004). Additionally, syncope was significantly more frequent among early presenters (22.8% vs. 6.6%, *P ═* 0.001). No significant differences were observed regarding age, sex, NIHSS score, stroke subtype, or most other clinical symptoms. These findings are summarized in Table 3.

**Table 3:** Comparison of clinical characteristics stratified by last known well time: ≤ 1 hour versus >1 hour

	**<1 hour** **Mean ± SD, *n* (%)**	**>1 hour** **Mean ± SD, *n* (%)**	***P***
N	60 (29.3)	145 (70.7)	
Age	71.72 ± 13.97	69.12 ± 13.11	0.214
Gender			0.335
female	23 (38.3%)	62 (42.8%)	
male	37 (61.7%)	83 (57.2%)	
Glasgow Coma Scale	13.60 ± 2.24	14.06 ± 1.54	0.217
NIHSS	8.32 ± 6.27	8.60 ± 6.47	0.846
Diabetes			0.029
No	43 (72.9%)	83 (57.6%)	
Yes	16 (27.1%)	61 (42.4%)	
3-month mRS outcome group			0.057
good	14 (35.9%)	11 (19.3%)	
poor	25 (64.1%)	46 (80.7%)	
Facial droop			0.004
No	50 (87.7%)	133 (97.8%)	
Yes	7 (12.3%)	3 (2.2%)	
Syncope			0.001
No	44 (77.2%)	127 (93.4%)	
Yes	13 (22.8%)	9 (6.6%)	
How did you get to the hospital?			<0.001
Direct ambulance (112)	49 (81.7%)	101 (69.7%)	
In-hospital transfer	3 (5.0%)	2 (1.4%)	
Intercity transfer	0 (0.0%)	34 (23.4%)	
Intracity transfer	4 (6.7%)	7 (4.8%)	
Walk-in	4 (6.7%)	1 (0.7%)	
First arrival at stroke center			<0.001
No	4 (6.7%)	41 (28.3%)	
Yes	56 (93.3%)	104 (71.7%)	

For each variable, percentages were calculated based on available data. Row-specific denominators varied, attributable to missing responses or incomplete follow-up data for specific questionnaire items. **Abbreviations**: mRS, modified Rankin Scale; N, number; NIHSS, National Institutes of Health Stroke Scale; SD, standard deviation.

### Comparison according to 2-hour arrival time

In the comparison of patients arriving within 2 h vs after 2 h, diabetes mellitus remained significantly associated with delayed hospital arrival (43.3% vs. 30.1%, *P ═* 0.039). Among presenting symptoms, facial droop was significantly associated with earlier hospital arrival (12.3% vs. 0.0%, *P* < 0.001). No statistically significant differences were found regarding age, sex, stroke severity, or most other clinical variables. Detailed comparisons are shown in Table 4.

**Table 4:** Comparison of clinical characteristics based on last known well time: ≤2 h versus >2 h

	**≤2 hours** **Mean ± SD, *n* (%)**	**>2 hours** **Mean ± SD, *n* (%)**	***P***
N	84 (41.7)	121 (58.3)	
Age	70.29 ± 13.94	69.60 ± 13.04	0.840
Gender			0.169
female	31 (36.9%)	54 (44.6%)	
male	53 (63.1%)	67 (55.4%)	
Glasgow Coma Scale	13.76 ± 2.02	14.03 ± 1.59	0.331
NIHSS	8.56 ± 6.50	8.49 ± 6.35	0.911
Diabetes			0.039
No	58 (69.9%)	68 (56.7%)	
Yes	25 (30.1%)	52 (43.3%)	
3-month mRS outcome group			0.052
good	17 (34.0%)	8 (17.4%)	
poor	33 (66.0%)	38 (82.6%)	
Facial droop			<0.001
No	71 (87.7%)	112 (100.0%)	
Yes	10 (12.3%)	0 (0.0%)	
Syncope			0.068
No	68 (84.0%)	103 (92.0%)	
Yes	13 (16.0%)	9 (8.0%)	
How did you get to the hospital?			<0.001
Direct ambulance (112)	67 (79.8%)	83 (68.6%)	
In-hospital transfer	4 (4.8%)	1 (0.8%)	
Intercity transfer	4 (4.8%)	30 (24.8%)	
Intracity transfer	5 (6.0%)	6 (5.0%)	
Walk-in	4 (4.8%)	1 (0.8%)	
First arrival at stroke center			<0.001
No	9 (10.7%)	36 (29.8%)	
Yes	75 (89.3%)	85 (70.2%)	
Did the emergency medical services (112) personnel inform you that thrombolytic therapy could be administered to the patient?			0.018
No	61 (95.3%)	75 (83.3%)	
Yes	3 (4.7%)	15 (16.7%)	

Percentages were derived from available data for each variable. Row-specific denominators varied due to missing responses or incomplete follow-up data for specific questionnaire items. **Abbreviations**: mRS, modified Rankin Scale; N, number; NIHSS, National Institutes of Health Stroke Scale; SD, standard deviation.

### Comparison according to 3-hour arrival time

When patients were grouped according to arrival within or beyond 3 h, facial droop remained significantly associated with early hospital presentation (10.9% vs. 0.0%, *P <* 0.001). Additionally, vision loss was more frequently reported among patients arriving later (6.9% vs. 1.1%, *P ═* 0.042). Syncope was also associated with earlier hospital arrival (16.3% vs. 6.9%, *P ═* 0.034). No significant differences were observed for demographic variables, stroke severity scores, or most other clinical features. These results are presented in Table 5.

**Table 5:** Clinical characteristics stratified by last known well time (≤3 h vs. >3 h)

	**≤3 hours** **Mean ± SD, *n* (%)**	**>3 hours** **Mean ± SD, *n* (%)**	***P***
N	99 (48.2)	106 (51.8)	
Age	70.57 ± 13.68	69.24 ± 13.15	0.542
Gender			0.157
female	37 (37.4%)	48 (45.3%)	
male	62 (62.6%)	58 (54.7%)	
Glasgow Coma Scale	13.92 ± 1.90	13.92 ± 1.66	0.999
NIHSS	8.14 ± 6.52	8.87 ± 6.29	0.352
Diabetes			0.170
No	64 (66.0%)	62 (58.5%)	
Yes	33 (34.0%)	44 (41.5%)	
3-month mRS outcome group			0.218
good	17 (29.8%)	8 (20.5%)	
poor	40 (70.2%)	31 (79.5%)	
Facial droop			<0.001
No	82 (89.1%)	101 (100.0%)	
Yes	10 (10.9%)	0 (0.0%)	
Vision loss			0.042
No	91 (98.9%)	94 (93.1%)	
Yes	1 (1.1%)	7 (6.9%)	
Syncope			0.034
No	77 (83.7%)	94 (93.1%)	
Yes	15 (16.3%)	7 (6.9%)	
Education group			0.038
higher	20 (35.7%)	13 (19.7%)	
lower	36 (64.3%)	53 (80.3%)	
How did you get to the hospital?			<0.001
Direct ambulance (112)	79 (79.8%)	71 (67.0%)	
In-hospital transfer	4 (4.0%)	1 (0.9%)	
Intercity transfer	5 (5.1%)	29 (27.4%)	
Intracity transfer	6 (6.1%)	5 (4.7%)	
Walk-in	5 (5.1%)	0 (0.0%)	
First arrival at stroke center			<0.001
No	11 (11.1%)	34 (32.1%)	
Yes	88 (88.9%)	72 (67.9%)	

Percentages were computed using the available data for each variable. Row-specific denominators varied due to missing responses or incomplete follow-up for specific questionnaire items. **Abbreviations**: mRS, modified Rankin Scale; N, number; NIHSS, National Institutes of Health Stroke Scale; SD, standard deviation.

### Comparison according to the 4.5-hour therapeutic window

Among patients presenting within vs after the 4.5-hour therapeutic window, hypertension was significantly more prevalent in the delayed arrival group (74.1% vs. 59.3%, *P ═* 0.020). Regarding presenting symptoms, facial droop showed a significant association with earlier hospital arrival (9.0% vs. 0.0%, *P ═* 0.003), whereas diplopia was more frequently observed in patients presenting later (3.7% vs. 0.0%, *P ═* 0.042). No other demographic or clinical variables exhibited significant intergroup differences. Detailed comparisons are presented in Table 6. Overall patterns of symptom presentation and vascular comorbidities associated with early vs delayed hospital arrival across various time thresholds are summarized in Figure 3.

**Table 6:** Comparison of clinical characteristics stratified by last known well time (≤4.5 h vs >4.5 h)

	**≤4.5 hours** **Mean ± SD, *n* (%)**	**>4.5 hours** **Mean ± SD, *n* (%)**	***P***
N	120 (58.5)	85 (41.5)	
Age	69.70 ± 14.22	70.13 ± 12.20	0.904
Gender			0.174
female	46 (38.3%)	39 (45.9%)	
male	74 (61.7%)	46 (54.1%)	
Glasgow Coma Scale	13.88 ± 1.84	13.98 ± 1.69	0.445
NIHSS	8.52 ± 6.46	8.51 ± 6.35	0.835
Hypertension			0.020
No	48 (40.7%)	22 (25.9%)	
Yes	70 (59.3%)	63 (74.1%)	
3-month mRS outcome group			0.258
good	18 (29.0%)	7 (20.6%)	
poor	44 (71.0%)	27 (79.4%)	
Facial droop			0.003
No	101 (91.0%)	82 (100.0%)	
Yes	10 (9.0%)	0 (0.0%)	
Vision loss			0.057
No	109 (98.2%)	76 (92.7%)	
Yes	2 (1.8%)	6 (7.3%)	
Diplopia			0.042
No	111 (100.0%)	79 (96.3%)	
Yes	0 (0.0%)	3 (3.7%)	
Education group			0.017
higher	24 (35.3%)	9 (16.7%)	
lower	44 (64.7%)	45 (83.3%)	
How did you get to the hospital?			<0.001
Direct ambulance (112)	96 (80.0%)	54 (63.5%)	
In-hospital transfer	4 (3.3%)	1 (1.2%)	
Intercity transfer	8 (6.7%)	26 (30.6%)	
Intracity transfer	7 (5.8%)	4 (4.7%)	
Walk-in	5 (4.2%)	0 (0.0%)	
First arrival at stroke center			<0.001
No	15 (12.5%)	30 (35.3%)	
Yes	105 (87.5%)	55 (64.7%)	

Percentages were calculated based on the available data for each variable; consequently, denominators for these calculations varied by row due to missing responses or incomplete follow-up data pertaining to specific questionnaire items. **Abbreviations**: mRS, modified Rankin Scale; N, number; NIHSS, National Institutes of Health Stroke Scale; SD, standard deviation.

**Figure 3.**
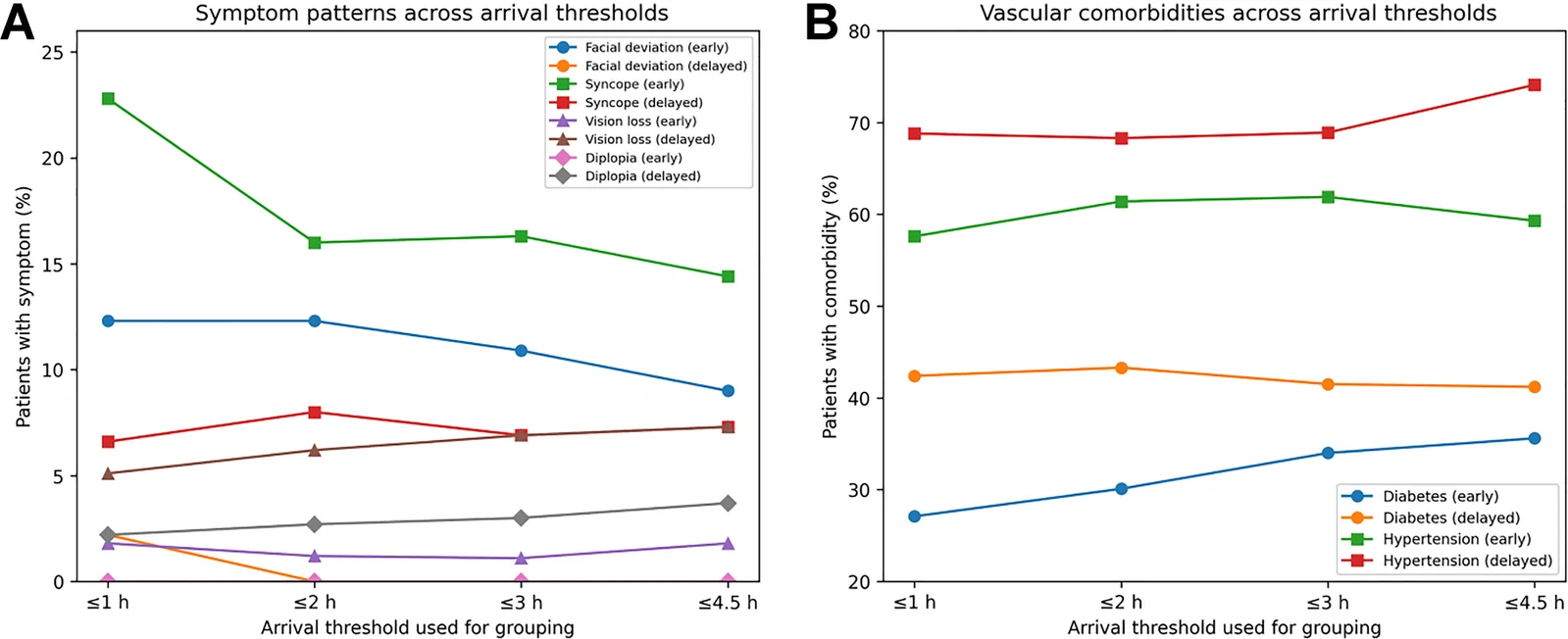
**Clinical characteristics associated with early versus delayed hospital arrival in acute stroke.** (A) Distribution of selected presenting neurological symptoms across specified arrival time thresholds (≤1 hour, ≤2 h, ≤3 h, and ≤4.5 h). Facial droop and syncope were more prevalent in patients presenting earlier. Conversely, visual symptoms, such as vision loss and diplopia, were more frequently observed among patients with delayed arrival. (B) Distribution of major vascular comorbidities stratified by early and delayed hospital arrival. Diabetes mellitus and hypertension were more prevalent in patients exhibiting delayed hospital arrival across multiple time thresholds.

### Association with 3-month functional outcome

Functional outcome at 3 months, assessed using the mRS, was analyzed in relation to clinical and prehospital variables. Three-month mRS follow-up data were available for 96 of the 205 included patients; data for the remaining 109 patients were unavailable. Patients with a favorable prognosis (mRS 0) were significantly younger than those with unfavorable outcomes (65.12 ± 11.50 vs. 73.17 ± 11.71 years, *P ═* 0.004). Although not statistically significant, patients with favorable outcomes tended to arrive at the hospital earlier (mean LKW time 198.0 ± 294.0 min vs. 314.4 ± 352.9 min, *P ═* 0.061). Findings related to altered consciousness were associated with poorer functional outcomes in the cohort (15.4% vs. 0%, *P ═* 0.031). No significant differences were observed concerning stroke subtype, comorbidities, or treatment modalities such as thrombolysis or thrombectomy. These findings are summarized in Table 7. The relationship between early hospital arrival and 3-month functional outcome is illustrated in Figure 4.

**Table 7:** Comparison of clinical variables and questionnaire responses stratified by 3-month mRS outcome (good vs poor prognosis)

	**good** **Mean ± SD, *n* (%)**	**poor** **Mean ± SD, *n* (%)**	***P***
N	25 (26%)	71 (74%)	
Age	65.12 ± 11.50	73.17 ± 11.71	0.004
Glasgow Coma Scale	14.12 ± 1.51	13.72 ± 1.95	0.354
NIHSS	8.43 ± 6.30	9.45 ± 6.79	0.713
Arrival within 1 hour			0.057
<1 hour	14 (56.0%)	25 (35.2%)	
>1 hour	11 (44.0%)	46 (64.8%)	
Arrival within 2 hours			0.052
<2 hours	17 (68.0%)	33 (46.5%)	
>2 hours	8 (32.0%)	38 (53.5%)	
Arrival within 3 hours			0.218
<3 hours	17 (68.0%)	40 (56.3%)	
>3 hours	8 (32.0%)	31 (43.7%)	
Arrival within 4.5 hours			0.258
<4.5 hours	18 (72.0%)	44 (62.0%)	
>4.5 hours	7 (28.0%)	27 (38.0%)	
Did the emergency medical services (112) personnel inform you about possible treatment options?			0.026
No	19 (100.0%)	47 (79.7%)	
Yes	0 (0.0%)	12 (20.3%)	
Did the emergency medical services (112) personnel inform you that thrombolytic therapy could be administered to the patient?			0.036
No	19 (100.0%)	48 (81.4%)	
Yes	0 (0.0%)	11 (18.6%)	
Altered consciousness			0.031
No	25 (100.0%)	55 (84.6%)	
Yes	0 (0.0%)	10 (15.4%)	

Percentages were calculated based on the available data for each variable. Row-specific denominators varied due to missing responses or incomplete follow-up data for specific questionnaire items. **Abbreviations**: mRS, modified Rankin Scale; N, number; NIHSS, National Institutes of Health Stroke Scale; SD, standard deviation.

**Figure 4.**
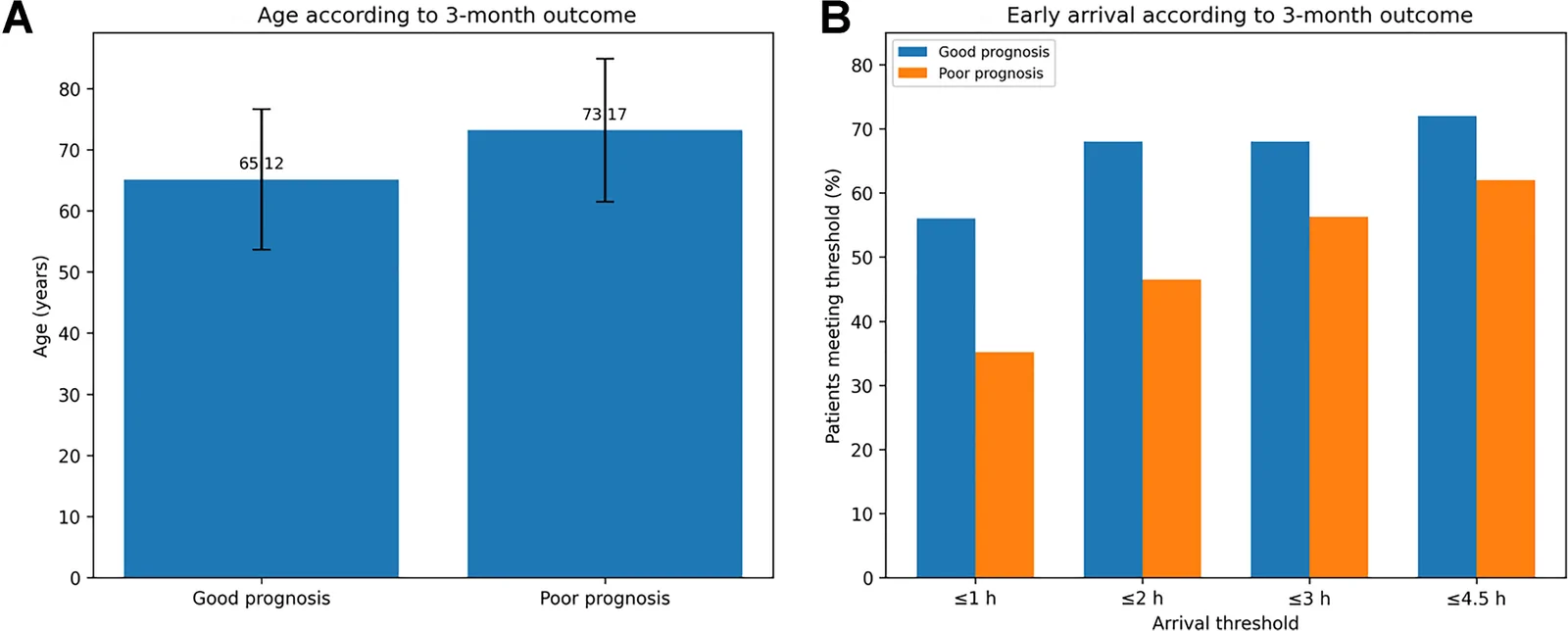
**Association between early hospital arrival and 3-month functional outcome.** (A) Mean age of patients stratified by 3-month functional outcome, defined as good prognosis (mRS score 0) or poor prognosis (mRS score 1–6). Patients with good prognosis were younger than those with poor prognosis; error bars represent SD. (B) Proportion of patients reaching the hospital within predefined early-arrival thresholds (≤1 h, ≤2 h, ≤3 h, and ≤4.5 h), stratified by 3-month functional outcome. Across all time thresholds, patients with good prognosis showed a numerically higher proportion of early hospital arrival than those with poor prognosis. **Abbreviations**: h, hours; mRS, modified Rankin Scale; SD, standard deviation.

## Discussion

This study investigated factors associated with delayed hospital arrival among patients experiencing a first-ever acute stroke, with a particular focus on the clinically critical 4.5-hour window from LKW to hospital presentation. Several key findings emerged. First, despite 58.5% of patients arriving within 4.5 h, only 29.3% reached the hospital within the first hour and 48.3% within 3 h, indicating persistent and substantial prehospital delay in a considerable proportion of patients. Second, diabetes mellitus was consistently associated with delayed presentation in the 1-hour and 2-hour analyses, while hypertension was more frequent among patients arriving after 4.5 h. Third, facial droop was repeatedly associated with earlier hospital arrival across multiple time thresholds, whereas visual symptoms such as vision loss and diplopia were more frequently linked to delayed presentation. Finally, younger age and the absence of altered consciousness were associated with better 3-month functional outcomes, and patients with a favorable prognosis also tended to exhibit shorter prehospital times.

Our finding that prehospital delay remains a significant barrier, despite advancements in stroke systems of care, aligns fully with existing literature. Extensive reviews have demonstrated that prehospital delay continues to account for the largest proportion of total treatment delay in acute stroke, often exceeding in-hospital inefficiencies [3]. Similarly, earlier research revealed that the majority of stroke patients historically failed to reach the emergency department within the most critical early treatment period, thereby limiting their eligibility for acute reperfusion therapies [4]. In our cohort, the mean LKW-to-arrival time was 338.66 ± 345.67 min, with only 41.0% arriving within 2 h, underscoring the ongoing relevance of this challenge in real-world stroke care.

These delays carry substantial clinical implications, as stroke represents one of medicine’s most time-sensitive emergencies. Saver’s seminal quantitative analysis estimated that the untreated ischemic brain loses approximately 1.9 million neurons per minute, powerfully reinforcing the biological imperative of every minute saved before reperfusion [1]. From this perspective, our observation that patients with a favorable 3-month outcome tended to arrive earlier than those with an unfavorable outcome (198.0 ± 294.0 vs. 314.4 ± 352.9 min, *P ═* 0.061) is directionally consistent with the “time is brain” paradigm, even though this difference did not achieve conventional statistical significance.

One of the most consistent exploratory observations in our study was the association between specific symptom patterns and faster hospital arrival. Facial droop was significantly associated with early presentation across the 1-hour, 2-hour, 3-hour, and 4.5-hour analyses (12.3% vs. 2.2%, *P ═* 0.004; 12.3% vs. 0.0%, *P* < 0.001; 10.9% vs. 0.0%, *P <* 0.001; and 9.0% vs. 0.0%, *P ═* 0.003, respectively). This likely reflects facial asymmetry as one of the most recognizable stroke warning signs for both bystanders and healthcare professionals. This interpretation aligns with studies demonstrating that symptom type strongly influences prehospital behavior, with more overt, alarming, or recognizable neurological deficits prompting earlier care-seeking [10, 11]. A Romanian tertiary-center study similarly found that facial palsy was negatively associated with late arrival, suggesting that visible focal deficits may facilitate faster symptom recognition and emergency response [13].

Conversely, visual symptoms in our study appeared to be associated with delayed presentation. Vision loss was more common in patients arriving after 3 h (6.9% vs. 1.1%, *P ═* 0.042), and diplopia was more frequent among those arriving after 4.5 h (3.7% vs. 0.0%, *P ═* 0.042). This pattern is clinically plausible. Visual symptoms may be perceived as unrelated to stroke, transient, ophthalmologic, or less urgent than motor deficits or speech disturbances. These findings may also reflect the recognized challenge of promptly identifying posterior circulation stroke manifestations, which are often less stereotypical and less rapidly recognized than anterior circulation stroke symptoms by both patients and prehospital care systems. Previous studies have similarly reported delayed recognition and treatment pathways in patients presenting with posterior circulation stroke-related symptom patterns [14]. Prior literature has also shown that symptom heterogeneity influences care-seeking, and that less typical or less dramatic symptom presentations can delay hospital arrival [10, 15]. This finding may have implications for public education campaigns, which often heavily emphasize Face–Arm–Speech–Time (FAST) symptoms but may underemphasize posterior circulation or visual manifestations.

Another exploratory observation was the higher frequency of reported syncope among early presenters, though this finding requires cautious interpretation as syncope is not a typical core manifestation of ischemic stroke. Syncope was significantly more common among early presenters in the 1-hour and 3-hour analyses (22.8% vs. 6.6%, *P ═* 0.001; 16.3% vs. 6.9%, *P ═* 0.034). While not a classical core stroke symptom, syncope is often perceived as a dramatic emergency event, which may heighten caregiver alarm and reduce hesitation in seeking medical attention. More broadly, prior studies have shown that severe or alarming symptom profiles, altered patient condition, and greater perceived urgency can shorten prehospital delay [9, 16]. As detailed imaging-based subtyping of syncopal presentations, including systematic evaluation of posterior circulation localization or basilar artery occlusion, was unavailable for this exploratory analysis, the observed relationship should not be interpreted as a stroke-specific mechanistic association. Rather, it may reflect the alarming nature of transient loss of consciousness, which could prompt earlier emergency response by patients or caregivers.

The relationship between vascular comorbidities and delayed arrival is also noteworthy. Diabetes mellitus was significantly more frequent in delayed presenters at the 1-hour and 2-hour thresholds (42.4% vs. 27.1%, *P ═* 0.029; 43.3% vs. 30.1%, *P ═* 0.039), while hypertension was more common in those arriving after 4.5 h (74.1% vs. 59.3%, *P ═* 0.020). These findings parallel recent meta-analytic evidence indicating that diabetes is associated with a higher risk of prehospital delay in acute ischemic stroke [15]. One plausible explanation is that patients with diabetes mellitus may experience metabolic symptoms related to hypo- or hyperglycemia that can partially mimic or obscure acute neurological deficits, potentially contributing to delayed recognition of stroke symptoms and postponement of emergency care-seeking behavior. Additionally, patients with chronic vascular disease may normalize certain neurological complaints, attribute symptoms to pre-existing illness, or delay decision-making due to symptom ambiguity. Previous literature has similarly suggested that metabolic symptom overlap may complicate timely stroke recognition in diabetic populations [17]. Alternatively, these comorbidities may cluster with older age, lower mobility, or more complex healthcare-seeking patterns. Although our study was not designed to establish causal mechanisms, the repeated association of diabetes with delayed arrival suggests that these patients may represent a target group for tailored awareness strategies.

Interestingly, we did not observe significant differences in age, sex, NIHSS score, or stroke subtype across most arrival-time thresholds, whereas some prior studies have identified older age, severe strokes, EMS use, education level, geography, distance, or socioeconomic factors as important determinants of delay [9, 18–20]. This discrepancy may reflect contextual differences in healthcare access, referral organization, local stroke awareness, sample size, or the composition of our cohort. It may also indicate that, in our setting, symptom perception and comorbidity profile exerted a more visible influence than broad demographic variables. Such heterogeneity across studies supports the view that prehospital delay is not driven by a single universal pathway but by an interplay of local, cultural, clinical, and logistical factors.

Our findings align with existing literature emphasizing modifiable prehospital barriers to timely stroke care. For instance, the Basel study identified low symptom awareness and suboptimal initial medical contact, particularly face-to-face visits to family physicians, as key modifiable contributors to delay [21]. Recent evidence from India, China, Africa, and a systematic review from Indonesia further underscore the importance of symptom recognition, transportation routes, the number of facilities approached, hospital distance, socioeconomic factors, and EMS activation [15–20]. While our current analyses focused on clinical and symptom-related correlates, our questionnaire-based study design supports the significance of analyzing modifiable healthcare-access behaviors within regional stroke populations.

From a systems perspective, our results highlight the ongoing need for optimizing the prehospital stroke pathway. Consensus guidelines advocate for public education, training of emergency personnel, prehospital code stroke activation, and rapid transport to stroke-ready centers as essential elements for reducing onset-to-door delay [7]. Reviews of prehospital workflow optimization further suggest that improvements in triage and transport strategies can decrease critical treatment times and enhance reperfusion efficiency, even if improvements in hard outcomes are less consistent [22]. Decision analyses have also demonstrated that prehospital destination protocols can significantly impact treatment opportunities, particularly when balancing access to IVT with routing to thrombectomy-capable centers [23]. Given that a substantial proportion of patients in our setting still missed early time thresholds, these system-level interventions remain highly pertinent.

This study has several limitations. First, its single-center design at a tertiary stroke center may limit generalizability to other regions, hospital types, or organizational structures. Second, some prehospital variables were gathered from patient relatives via structured interviews, potentially introducing recall bias, particularly concerning symptom onset details and decision-making processes. Third, although the study collected a broad range of clinical and questionnaire-based variables, it may not have fully captured all social, cultural, geographic, and behavioral determinants of delay. Important potentially influential variables, such as exact residential distance to the hospital, real-time traffic conditions, detailed household income, caregiver socioeconomic characteristics, and standardized stroke-awareness measurements, were not systematically collected. These unmeasured factors may have contributed to variability in prehospital delay patterns and healthcare-seeking behavior. Fourth, subgroup analyses across multiple time thresholds may have reduced statistical power for certain comparisons. Additionally, some questionnaire-based variables relied on relative-reported information regarding prehospital events, which could introduce recall bias or response variability despite the use of structured interviews. The study primarily employed threshold-based unadjusted comparative analyses rather than predefined multivariable modeling approaches, meaning residual confounding may have influenced some observed associations. Furthermore, multiple comparisons were performed across several predefined arrival thresholds and clinical variables without formal adjustment for multiplicity, suggesting that some borderline statistically significant findings may be due to chance. Lastly, while syncope was recorded as a presenting feature based on prehospital clinical history, detailed imaging-based characterization of syncopal presentations, including posterior circulation localization or basilar artery occlusion status, was unavailable, thus requiring cautious interpretation of this finding. The strict dichotomization of mRS 0 vs 1–6 may limit comparability with conventional stroke outcome analyses using functional independence thresholds such as mRS 0–2. Finally, as an observational study, the identified associations should not be interpreted as causal.

Future studies should expand upon these findings using multicenter designs, larger sample sizes, and more granular prehospital pathway data. Subsequent investigations could also benefit from detailed subtype-specific analyses evaluating potential differences in prehospital delay patterns, symptom recognition, vascular risk factor distribution, stroke severity, treatment eligibility, and clinical outcomes across distinct acute stroke subtypes, including cardioembolic stroke, lacunar infarction, atherothrombotic stroke, and intracerebral hemorrhage. Prospective regional stroke registries incorporating EMS activation, initial medical contact behavior, interfacility transfer patterns, and detailed geographic variables would help identify locally actionable mechanisms of delay. Public health interventions should specifically target easily overlooked symptom profiles, such as visual complaints, and high-risk patient groups, including those with diabetes or hypertension. Educational efforts should address not only stroke recognition but also the necessary action pathway: immediate EMS activation and direct transport to stroke-capable centers. Finally, integrating structured prehospital education into undergraduate medical training, as initiated in this study through final-year medical student participation, may offer an additional long-term strategy to improve stroke systems awareness. Future multicenter investigations employing predefined multivariable regression models or continuous time-to-arrival analytical frameworks may further clarify the independent contribution of clinical and prehospital variables associated with delayed stroke presentation. Additional future research directions may include evaluating region-specific organizational stroke pathways, optimizing EMS triage, conducting public awareness intervention studies, examining behavioral determinants of delayed healthcare-seeking, and developing artificial intelligence-supported predictive models to identify patients at high risk for delayed presentation. Broader multicenter and international collaborative studies could further enhance understanding of how healthcare infrastructure, sociocultural factors, and stroke subtype variability influence prehospital stroke delay patterns. Moreover, future studies with larger cohorts and more balanced subgroup structures may benefit from continuous time-to-arrival analytical approaches, including log-transformed or nonparametric temporal regression models specifically designed for highly skewed prehospital delay distributions.

## Conclusion

Prehospital delay remains a significant barrier to timely acute stroke care in our cohort. Specific sociodemographic, clinical, and organizational factors influence timely hospital arrival after stroke. Improved recognition of stroke symptoms, optimized prehospital pathways, and increased public awareness can contribute to reducing delays in access to acute stroke care. These exploratory findings suggest the potential value of locally tailored strategies aimed at enhancing recognition of both typical and atypical stroke symptoms, strengthening EMS-centered referral behavior, and targeting modifiable barriers in vulnerable patient groups.

## Data Availability

The dataset generated and analyzed in this study is publicly available on the Istinye University Dataset Sharing Platform. Anonymized clinical data can be accessed at the following link: https://dataset.istinye.edu.tr/dataset?did=88. All data were fully anonymized in accordance with ethical regulations. Access is provided for research purposes through a controlled-access system under the platform’s standard licensing and data-sharing policies.
